# Treadmill training of rats after sciatic nerve graft does not alter accuracy of muscle reinnervation

**DOI:** 10.3389/fneur.2022.1050822

**Published:** 2023-01-19

**Authors:** Mohammed Barham, Jonas Andermahr, Henryk Majczyński, Urszula Sławińska, Johannes Vogt, Wolfram F. Neiss

**Affiliations:** ^1^Department II of Anatomy, University of Cologne and University Hospital of Cologne, Cologne, Germany; ^2^Kreiskrankenhaus Mechernich, Mechernich, Germany; ^3^Nencki Institute of Experimental Biology, Polish Academy of Sciences, Warszawa, Poland; ^4^Cluster of Excellence for Aging Research (CECAD) and Center of Molecular Medicine Cologne (CMMC), University of Cologne, Cologne, Germany; ^5^Department I of Anatomy, University of Cologne and University Hospital of Cologne, Cologne, Germany

**Keywords:** motoneuron, sciatic nerve, nerve regeneration, misdirected reinnervation, physical exercise, recovery of function

## Abstract

**Background and purpose:**

After peripheral nerve lesions, surgical reconstruction facilitates axonal regeneration and motor reinnervation. However, functional recovery is impaired by aberrant reinnervation.

**Materials and methods:**

We tested whether training therapy by treadmill exercise (9 × 250 m/week) before (run–idle), after (idle–run), or both before and after (run–run) sciatic nerve graft improves the accuracy of reinnervation in rats. Female Lewis rats (LEW/SsNHsd) were either trained for 12 weeks (run) or not trained (kept under control conditions, idle). The right sciatic nerves were then excised and reconstructed with 5 mm of a congenic allograft. One week later, training started in the run–run and idle–run groups for another 12 weeks. No further training was conducted in the run–idle and idle–idle groups. Reinnervation was measured using the following parameters: counting of retrogradely labeled motoneurons, walking track analysis, and compound muscle action potential (CMAP) recordings.

**Results:**

In intact rats, the common fibular (peroneal) and the soleus nerve received axons from 549 ± 83 motoneurons. In the run–idle group, 94% of these motoneurons had regenerated 13 weeks after the nerve graft. In the idle–run group, 81% of the normal number of motoneurons had regenerated into the denervated musculature and 87% in both run–run and idle–idle groups. Despite reinnervation, functional outcome was poor: walking tracks indicated no functional improvement of motion in any group. However, in the operated hindlimb of run–idle rats, the CMAP of the soleus muscle reached 11.9 mV (normal 16.3 mV), yet only 6.3–8.1 mV in the other groups.

**Conclusion:**

Treadmill training neither altered the accuracy of reinnervation nor the functional recovery, and pre-operative training (run–idle) led to a higher motor unit activation after regeneration.

## 1. Introduction

The ultimate goal of nerve repair is the morphological and functional recovery of the motor and sensory systems. Numerous investigations were conducted in either the sciatic nerve or the common fibular [peroneal] nerve to estimate the quality and quantity of regeneration and recovery of function using retrograde neuronal labeling, stereology, and electrophysiology (1–13), walking track measurements (5, 13–18), and video analysis of gait methodologies (10, 19–22).

Accuracy of motor and sensory reinnervation remains a major challenge after peripheral nerve transection. The regenerating axons tend to re-grow incorrectly and thus miss the original target (23, 24). Although motoneurons preferentially reinnervate motor nerves (25, 26), they do not reinnervate the motor endplates of the original muscle fibers. This misdirection of reinnervation in cranial and spinal nerves dramatically changes the myotopic organization of central motor nuclei (3, 4, 27–34). This results in an auto-paralytic syndrome (24, 35, 36) or other permanent functional deficiencies (3, 4, 35, 37, 38). Since the pioneering work of Otto von Büngner (39), there is considerable, yet contradictory, evidence that rehabilitation after peripheral nerve injury by *adequate* exercise may contribute to functional recovery (40, 41).

Several researchers have reported a positive effect of training following nerve crush or injury. This training included both an elevated source of drinking water (17, 39, 40, 42, 43) and balance and coordination training (44–46). However, no significant training effect on sensorimotor function was observed after sciatic nerve crush followed by endurance training (47). A detrimental training effect was even reported by van Meeteren et al. (48).

Following *nerve axotomy*, a beneficial training effect has been described (49–53). No training effect using forearm stimulation or whole-body vibration was seen by Sinis et al. (54) and de Oliveira Marques et al. (55), respectively. A *negative training effect*, that is, better recovery of the SFI (sciatic functional index) in idle than in trained rats was reported by Rustemeyer et al. (56).

Treadmill training has been used in several previous studies of sciatic nerve transection (45, 52) or sciatic nerve graft (56). This investigation differs from those markedly in two ways: First, no one has previously studied the training effect on healthy animals before a nerve lesion. Does better fitness at the onset of neuronal regeneration make a difference in recovery? Second, we have trained rats until 13 weeks after surgery; only two other investigations report a similar duration of treadmill training. Rustemeyer et al. (56) trained rats for 16 weeks after sciatic nerve graft but only animals with FK506 therapy. Maqueste et al. (49) trained for 10 weeks after peroneal nerve suture but focused on axonal regeneration of mechano- and metabosensitive muscle afferents. For these reasons, this study was designed to analyze the effects of treadmill training on the onset of neuronal regeneration in healthy animals before a nerve lesion and the effects of prolonged treadmill training on functional recovery after sciatic nerve graft.

## 2. Materials and methods

### 2.1. Experimental design

All experimental procedures were performed according to the guidelines of the European Union Council (86/609/EU), and all experimental protocols were approved by the Local Animal Protection Committee (Bezirksregierung Köln, Az. 50.203.2-K35, 34/2001). Thirty-two adult, female, and Lewis rats (175–200 g; LEW/SsNHsd, Harlan, Bicester, England) were used for experimentation, and eight age-matched, male Lewis rats were used as congenic nerve donors. Throughout the study, all animals were fed standard laboratory food (Ssniff, D-59494 Soest, Germany) and tap water *ad libitum*. All animals were maintained on an artificial light–dark cycle of 12 h on and 12 h off.

The experimental animals were divided into four groups of eight rats each: *run–run, run–idle, idle–run*, and *idle–idle*. All groups received identical surgery but different physical exercises. They either received treadmill training (run) or no training (idle) for 12 weeks before and/or 12 weeks after the nerve transplant, starting 1 week after surgery. During the 12-week training, walking track analyses were performed to assess the differences in recovery of function. The evoked compound muscle action potential (CMAP) was recorded directly before nerve transection and once again 13 weeks post-transplantation. After the second set of CMAP recordings, retrograde fluorescent labeling was applied to count the number of regenerated sciatic motoneurons and assess the accuracy of reinnervation.

### 2.2. Physical exercise before surgery

Sixteen experimental rats (run–run and run–idle groups) were trained on a 5-lane treadmill (Panlab LE 8710R; www.panlab.com) for 12 weeks. During the first 4 weeks, each training session (two times per day at 08.00 and 16.00; nine sessions/week) began at a speed of 3 m/min that was gradationally increased to 6, 9, 12, 15, and 18 m/min, and then decreased by the same increments. The animals ran 75 m/session during the first week, 100 m during the second, 125 m in the third week, and 150 m during the fourth week. During weeks 5–12, the animals ran 250 m/session (9 × 250 m/week), reaching up to 30 m/min. All rats adapted to the treadmill well (see results). Pending the 12 weeks, the other 16 experimental rats (idle-run and idle–idle groups) and the eight age-matched donor rats were kept sedentary. Namely, two rats per cage were kept–and allowed to ambulate—in type IIIH cages [L425 mm x W265 mm x H180 mm with 800 cm^2^ floor area (Bioscape, Rauxel, 44579 Germany)] in the same animal room as the rats in training.

### 2.3. Sciatic nerve graft

The microsurgery was performed under an operating microscope (Carl Zeiss) after an intraperitoneal injection of 0.05 ml Ketanest/Rompun per 10 g body weight [100 mg Ketanest (WDT, D-30827 Garbsen, Germany) plus 10 mg Rompun (Bayer AG, 51368 Leverkusen, Germany) per kg body weight, that is, 1.0 ml Ketanest 100 mg/ml plus 0.5 ml Rompun 20 mg/ml mixed with 3.5 ml NaCl 0.9%]. In all 32 experimental rats, the CMAP of the sciatic nerve was measured on both the left and the right sides. On the right experimental side, after exposure of the sciatic nerve, a 5-mm long segment of the nerve was excised between the greater trochanter and the sciatic trifurcation. The continuity of the nerve was reconstructed with a 5-mm long fresh sciatic nerve graft from an age-matched, congenic donor rat by applying two 11–0 atraumatic sutures (Ethicon EH 7438G, D-22851 Norderstedt, Germany). For reconstructive surgery, each donor rat was anesthetized (see above), and four 5-mm long segments, that is, two of the left and two of the right sciatic nerve were successively excised and later implanted into four experimental animals. After the excision of the nerve grafts, the donor rats were immediately euthanized by cervical dislocation.

LEW/SsNHsd is an inbred rat strain in which congenic heterologous transplants behave like autologous transplants. Neither in this nor in a previous study (5), we have encountered immune reactions up to 12 weeks after the sciatic nerve transplant. Similarly, automutilation did not occur in the LEW/SsNHsd rats (16, 57, 58).

### 2.4. Physical exercise after surgery

The run–idle and idle–idle groups were kept sedentary for 13 weeks of axonal regeneration. The run–run and idle-run groups were also kept sedentary for 1-week post-operation to allow for undisturbed wound healing. Then, training was resumed in run–run group (9 × 250 m/week) and was initiated in idle-run group for 12 weeks.

The rats with preoperative training promptly started running again and did not require reinforcement by the electric grid. Training of the idle-run rats was started gradually, exactly as the training in the run–run and run–idle groups before the surgery. All training sessions were closely supervised by Dr. Barham as the veterinarian, and animals that occasionally showed signs of fatigue were excepted from the session.

### 2.5. Walking track analysis

To estimate the functional recovery of gait, we have analyzed walking tracks strictly identical as described by Barham et al. (5). We used the program FOOTPRINTS [version 1.22, free university license (59)] for measuring the walking tracks and FOOTPRINTS STATISTICS (Robert A. Neiss; version 13-1, free license: neiss.anatomie@uni-koeln.de) for mathematical processing.

The Sciatic Functional Index (SFI) (14, 60) of each rat was calculated from walking tracks obtained 10 times before injury (normal values), immediately after nerve graft (paralysis values), and then a further 12 times post-operation (recovery values). For the recording of walking tracks, we used a 1-m long wooden ramp (30° inclination) with a small dark chamber at the top end. The track was covered with a white paper strip. The plantar surfaces of both hind paws were painted with a solution of black food coloring (mixture of European food colors E135, E104, E110, and E125) made sticky with powdered sugar (15 ml of food coloring + 120 g of sugar powder), and then the rat walked or in the case of lesioned animals rather crawled up the ramp into the top chamber. Footprints of hind paws left a stark contrast on white paper (21, 61); one strip contained seven to eight prints of each paw. The walking tracks were digitized, binarized, and converted to a resolution of 75 dpi as required for FOOTPRINTS.

Using FOOTPRINTS Print Length (PL), Total Spreading (TS, distance from the first to the fifth toe) and Intermediary Spreading (IS, distance from the second to the fourth toe) were measured for each normal (N) left and each experimental (E) right footprint (see Supplementary material 1). For each track, the mean value of 7–8 footprints of the normal side (NPL, NTS, and NIS) and the mean of the experimental side (EPL, ETS, and EIS) were calculated. In addition, the most important factor of the SFI (62), the normal step length, was measured from the left to the right, i.e., the distance from a stance on the normal left hind limb to the forward movement of the experimental right hind limb [Normal To Opposite Foot (NTOF)], and the experimental step length was measured from right to left [Experimental To Opposite Foot (ETOF)]. The lesioned hind limb is weakened and cannot support the body weight during stance as well as the normal hind limb, resulting in an often much smaller ETOF than NTOF. ETOF minus NTOF is a direct measure of limping. The maximal values of NTOF and ETOF of each track were used for the SFI, not the mean (62). Using the average of means of eight tracks, the Sciatic Functional Index (14) was calculated as follows:


$$\begin{array}{r} SFI=(\frac{ETOF-NTOF}{NTOF}+\frac{NPL-EPL}{EPL}+\frac{ETS-NTS}{NTS}\\ +\frac{EIS-NIS}{NIS})÷4\;\times \;220 \end{array}$$

The measurements of ~4,100–4,700 footprints of the 616 walking tracks were carried out by specially trained technicians. To prevent observer bias, all mathematical processing was performed by another person, and only after raw data collection of walking tracks and the counting of labeled motoneurons on coded sections had been completed.

### 2.6. Motor nerve conduction test

Using Neuropack 2 (www.nihonkohden.com), the evoked compound muscle action potential of the soleus muscle was measured on both sides first, immediately before nerve graft (normal CMAP) and second, directly before neuronal labeling (regenerated/contralateral CMAP). Under Ketanest/Rompun anesthesia, the sciatic nerve was exposed at the mid-thigh through a gluteal muscle-splitting incision. A muscle pouch was formed and filled with 3–4 ml of paraffin oil as an electrical isolator. Then, a bipolar needle electrode was inserted into the soleus muscle laterally bypassing the gastrocnemius muscle [see Figures 100, 101 in Greene (63)]. Great care was taken that both uninsulated tips of the electrode had no contact with the gastrocnemius muscle but exclusively positioned in the soleus muscle. A flush-tip monopolar stimulator was placed onto the sciatic nerve directly proximal to the transplant, 5 mm distal to the spine on the control side. The duration of the stimulus was 0.2 ms at the supramaximal intensity of 2.6 mA. The recording analysis time was 20 ms with 10–1,000 μV sensitivity and filters of 20–3,000 Hz. We measured the amplitude (the peak-to-peak height of the main evoked electromyography waveform, excluding late waves) and latency of muscle contraction (delay of the peak wave). All measurements were repeated 10 times.

### 2.7. Retrograde fluorescent neuronal labeling

**Figure 1:**
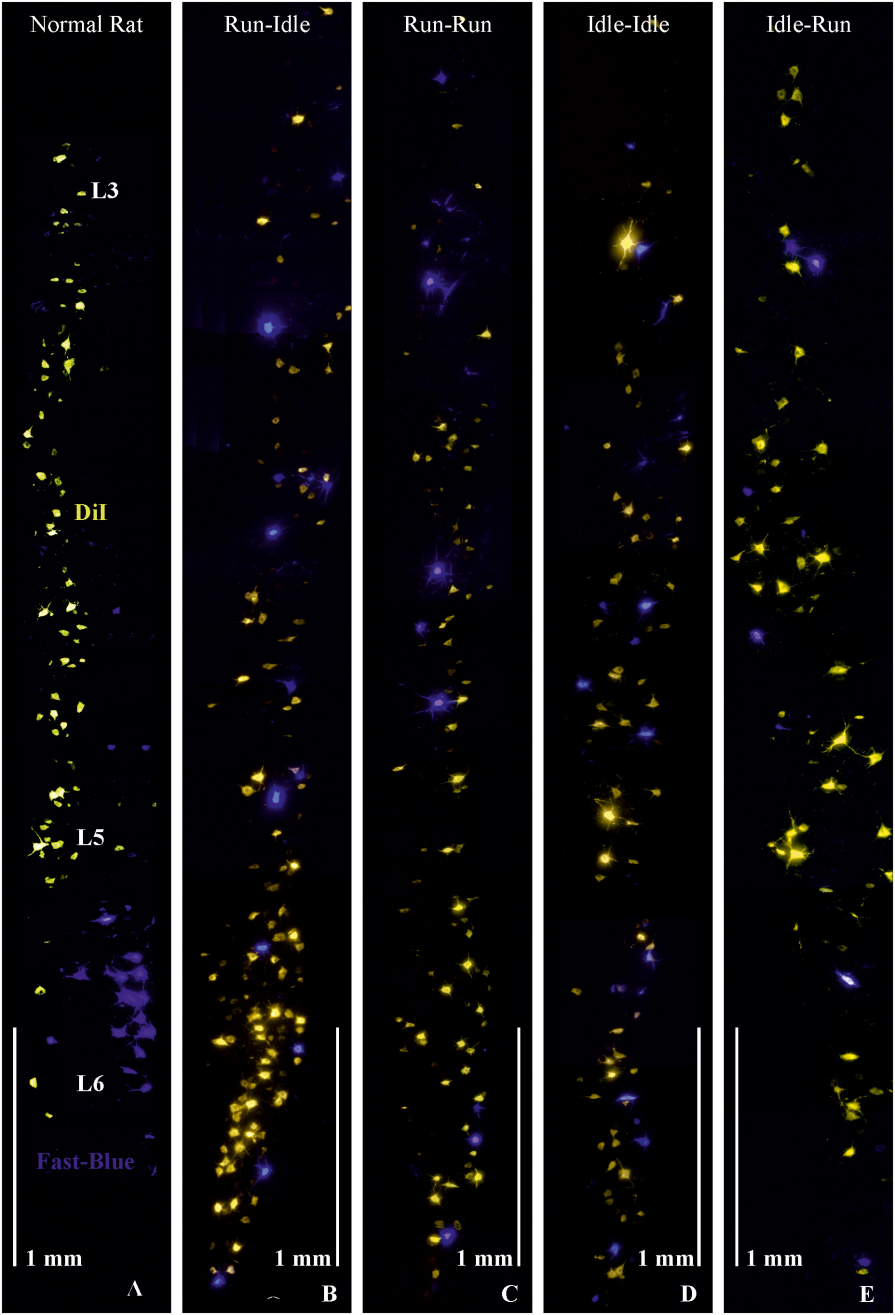
Retrograde labeling of motoneurons in the lumbar spinal cord. **(A)** Normal rat, without graft transplantation, shows highly ordered columns of motoneurons of dorsiflexor muscles (*yellowish-orange*) retrogradely labeled by DiI applied to the common fibular nerve and plantar flexor motoneurons (*blue*) retrogradely labeled by Fast-Blue (FB) applied to the soleus nerve, indicating correct myotopic organization of motoneurons in the ventral (anterior) horn of the lumbar spinal cord (L3-L6). **(B–E)** These four images demonstrate chaotic and randomly misdirected regeneration of the common fibular and soleus nerves and complete loss of lumbar myotopy in all groups that received a 5-mm allograft nerve transplant. Note the random salt and pepper distribution of the retrogradely labeled motoneurons innervating the plantar flexor (blue labeling) and dorsiflexor (yellow labeling) muscles. Objective 10×, scale bar 1 mm.

Thirteen weeks post-transplantation, sufficient time for sciatic nerve regeneration and muscle reinnervation in rats (1, 3–5), the final walking track was recorded. The animals were re-anesthetized and the second set of CMAP data was recorded on the left and right sides. In addition, on the right side, the common fibular (peroneal) nerve was transected 10 mm proximal to the fibular head, and the soleus branch of the tibial nerve was close to its entrance into the soleus muscle. A few crystals of DiI (1,1′-Dioctadecyl-3,3,3′,3′-Tetramethylindocarbocyanine Perchlorate; Molecular Probes, The Netherlands; cat, no, D-282) were applied to the proximal stump of the common fibular nerve and some crystals of Fast-Blue (FB; EMS-Chemie, D-64823 Groß-Umstadt, Germany) were applied to the proximal stump of the soleus nerve. Both fluorescent tracers were always applied distal to the transplant site. Care was taken to avoid bleeding and thus blood diffusion of the fluorescent dyes. Figure 1 (intact rat) shows that cross-diffusion of tracers did not occur. Fourteen days later, all rats were transcardially perfused with 4% formaldehyde in 0.1M phosphate buffer of pH 7.4. The lumbosacral spinal cord [L2-S2; the rat sciatic nerve that originates from L4-L6 (64)] was removed and postfixed overnight at 4°C by immersion in the same fixative. The spinal cord was longitudinally cut into a complete series of 36–40 vibratome sections of 50 μm thickness (Leica VT 1000-S), as the fibular communal nerve nucleus and the soleus nerve nucleus encompass 22–30 sections of 50 μm thickness. Then, all sections were collected, mounted, air-dried, and stored in the dark at 4°C until microscopy. Images for neuron counting (1,300 × 1,030-pixel, 3.8 MB) were recorded within the first 24 h after cutting with a Zeiss Axioskop plus ProgResC14 CCD camera (D-07743 Jena, Germany, www.jenoptik.com), using a Zeiss Plan-Neofluar 10x.

Sections were observed through shift-free AHF filter sets: www.ahf.de F11-013 for Fast Blue (FB; blue) and F41-003 for DiI (yellow/orange). Two TIFF images were recorded per field of view. The full length of the labeled motoneuron columns was covered by tiling the image frames. Using Image-Pro Plus 6.3 (www.mediacy.com: Rockville, Maryland USA 20852) and employing the physical fractionator (65), all retrogradely labeled motoneurons with a visible cell nucleus were counted in every second section (66), throughout the spinal cord. The counting of labeled neurons was performed on the operated side in separate and superimposed image files (4, 67). For a detailed discussion of the stereology, see Note on cell counting in: Valero-Cabré et al. (3).

### 2.8. Statistical evaluation

All values are expressed as the mean ± standard deviation. Separate one-way ANOVA tests were used to analyze the effect of the training groups on the number of spinal motoneurons innervating the dorsiflexor muscles (DiI-label), respectively, the plantar flexor muscle (FB-label) of double-labeled motoneurons (DiI + FB label), as well as the total number of labeled neurons per animal. The training effects on SFI and of the motor nerve conduction tests were analyzed with a repeated measure two-way ANOVA with two factors: (1) Pre- and post-nerve graft and (2) type of training, followed by *post hoc* Fisher's LSD tests. In all tests, *p* < 0.05 was considered significant.

## 3. Results

### 3.1. General observations on treadmill training

All rats, those with training before nerve injury (run–run and run–idle groups), as well as those with training exclusively after nerve transection and repair (idle-run group), were accustomed to the treadmill. Most of the rats required a single contact with the electric grid before perpetually running on the treadmill during subsequent sessions (except when exhausted, see below). When lifted to the treadmill, the animals jumped rather eagerly into their lanes. The distance of 250 meters per track was performed at a maximal 30 m/min, that is, ~11-min run, including a lower speed of warm-up, a procedure, which was manageable for all rats. However, after two sessions per day, a few trained rats showed signs of exhaustion. These signs included ceasing to run and resting on the disconnected electric grid, especially in the afternoon sessions. With 16 rats in training, that is, 16 × 9 runs = 144 training runs per week, this occurred to ~3–5 rats per week. These rats were immediately returned to the cage, and in all cases of exhaustion, they resumed running—without problems—on the following day. In daily controls by a veterinarian specialist in experimental animal husbandry, exercised rats seemed less prone to obesity and appeared more vigilant than idle animals.

### 3.2. Regeneration of motoneurons

Ninety-one days after the nerve graft, the animals were reoperated for the placement of electrodes for the second set of motor nerve conduction measurements (see Section 3.4), and subsequently the application of neuronal tracers. During this surgery, no signs of neuroma formation were observed at or distal to the site of the nerve graft.

As in our previous studies (3–5), we retrogradely labeled the transected soleus nerve with Fast Blue (FB) and the cut common fibular nerve with DiI. Hereby, we stained all spinal motoneurons innervating the soleus muscle as an important plantar flexor (FB blue labeling in Figure 1), while DiI (yellow/orange labeling in Figure 1) labeled the motoneurons of most dorsiflexor muscles of the foot and toes (anterior tibialis, extensor digitorum longus and brevis, and extensor halluces longus muscles).

In normal female Lewis rats, 443 ± 74 motoneurons projecting through the common fibular nerve innervating the dorsiflexor muscles, and 106 ± 23 motoneurons projecting through the soleus nerve innervating the plantar flexor muscles can be labeled by this tracer application (5). In all operated groups, the number of dorsiflexor motoneurons dropped to ~350–380, while that of motoneurons innervating soleus muscle decreased to ~85–125 (details see Table 1). In addition, we counted 37 ± 28 double-labeled neurons per rat in the run–idle group but only 10–15 in the other three experimental groups (Table 1). Such double-labeled neurons simultaneously innervate both dorsiflexor and plantar flexor muscles, that is, antagonistic muscles were never observed in normal, non-operated animals—only after nerve injury and regeneration. The change in neuron numbers after nerve lesion and regeneration coincides with dramatic changes in the myotopic organization of neurons.

**Table 1:** Number of retrogradely labeled spinal motoneurons.

**Animal groups**	**DiI-labeled neurons innervating dorsiflexor muscles**	**FB-labeled neurons innervating plantar flexor muscles**	**Double-labeled neurons**	**Total (DiI+FB)**	**Number of neurons (% of normal count)**
Normal rats^*^	443 ± 74	106 ± 23	Zero	549 ± 83	100%
Run–idle	352 ± 139	126 ± 53	37 ± 28	515 ± 193	93.9%
Run–run	379 ± 99	86 ± 36	10 ± 5	476 ± 99	86.6%
Idle–idle	364 ± 54	101 ± 39	15 ± 10	480 ± 94	87.4%
Idle–run	352 ± 68	85 ± 23	10 ± 7	453 ± 76	82.4%

DiI-labeled motoneurons of the dorsiflexor muscles projecting through the common fibular nerve and Fast Blue-labeled motoneurons of the plantar flexor muscles projecting through the soleus nerve in untreated animals and rats that had or had not received 12 weeks of treadmill-training before and/or after sciatic nerve graft (mean ± SD). Double-labeled motoneurons innervating dorsiflexor and plantar flexor muscles at the same time (mis-sprouting) were found in all rats that had received nerve surgery but not in intact animals. The right column shows the total number of motoneurons that were regenerated 91 days after nerve surgery.

* Our previous data from six normal Lewis rats (5).

In non-operated rats, the perikarya of motoneurons of the dorsiflexor muscles projecting through the common fibular nerve are localized in the lumbar segments L3–L5 as a cylinder-shaped nucleus in the ventral horn close to the white matter. The perikarya of plantar flexor motoneurons projecting through the soleus nerve are found in lumbar segments L5–L6, as a shorter column, more ventrally overlapping the motoneurons of the dorsiflexor muscles (Figure 1, *normal rat*).

In all operated rats—regardless of the training modality—the myotopic organization was replaced by a chaotic innervation, as previously shown in tracing studies of the regenerated sciatic nerve (3–5). Due to a misdirection of reinnervation, rats of all experimental groups showed evidence of a complete loss of myotopic localization of flexor and extensor motoneurons in the anterior horn of the spinal cord (Figure 1). There was no difference in the number and localization of misinnervating neurons among the run–idle, run–run, idle–idle, and idle-run groups. Separate one-way ANOVA tests showed no significant differences among all training types regarding the number of (1) DiI-labeled motoneurons, (2) FB-labeled motoneurons, and (3) the total number of labeled neurons per animal.

In summary, neither the numbers (Table 1) nor the distribution of labeled motoneurons along with the lumbar spinal cord (Figure 1) indicates any effect of treadmill training on the morphological outcome of neural regeneration.

### 3.3. Recovery of gait

**Figure 2:**
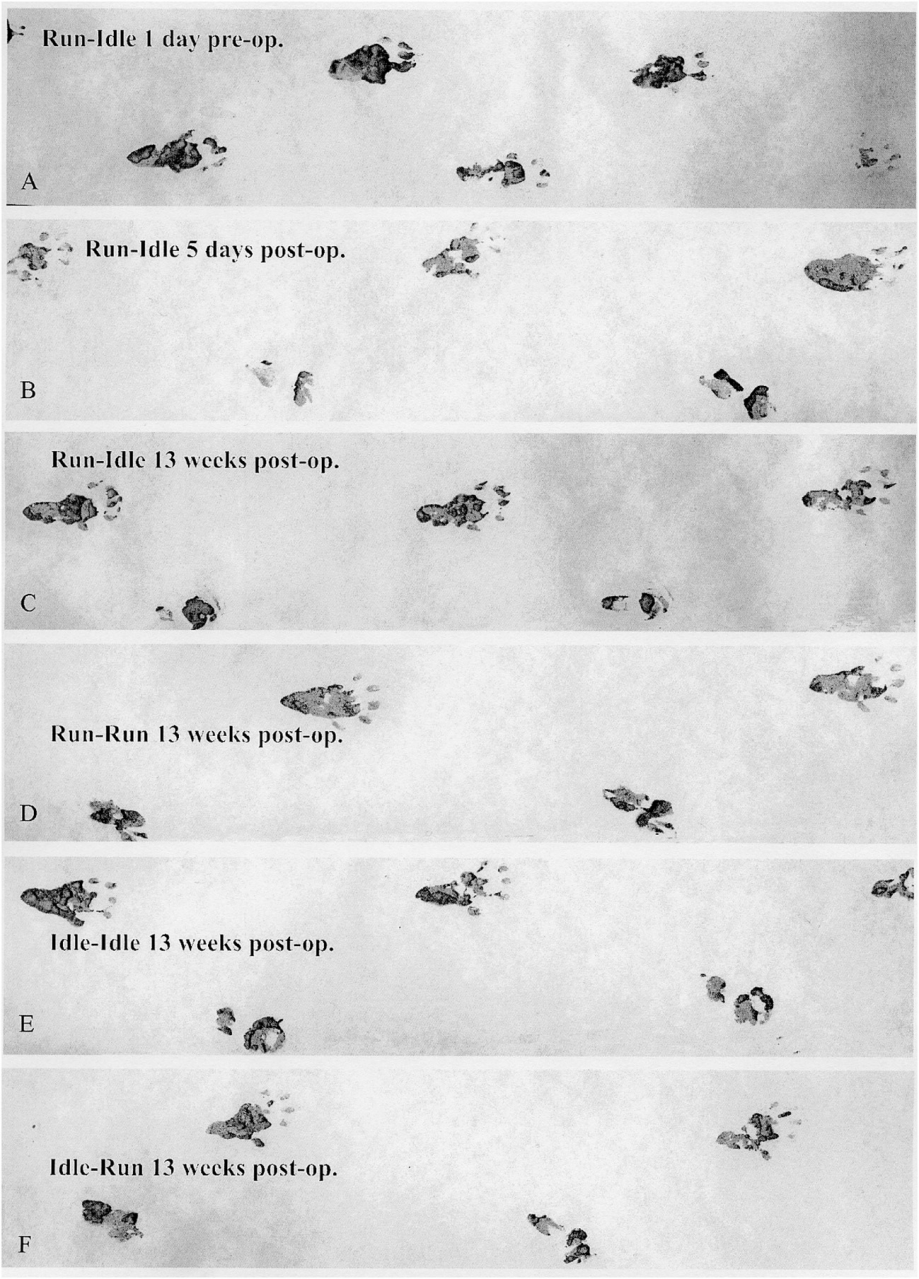
Walking track analysis. Representative sets of footprints running from left to right. **(A)** This track was made by a trained normal rat (run–idle 1 day before nerve transplant), with a symmetrical gait and well-splayed toes. **(B)** The second track presents the completely paralyzed right (operated) hind paw 5 days after the sciatic nerve transplant. The four lowest tracks **(C–F)** were recorded 13 weeks after sciatic nerve surgery. The upper prints from the second to the last track stem are from the intact left hind paw, and the lower are prints from the misdirectly reinnervated right hind paw. A comparison of the run–idle tracks at 5 and 91 days after nerve transplantation shows no improvement in the motor control of the right foot and the gait pattern. For details on the measurement of footprints, see Supplementary Figure 1.

**Figure 3:**
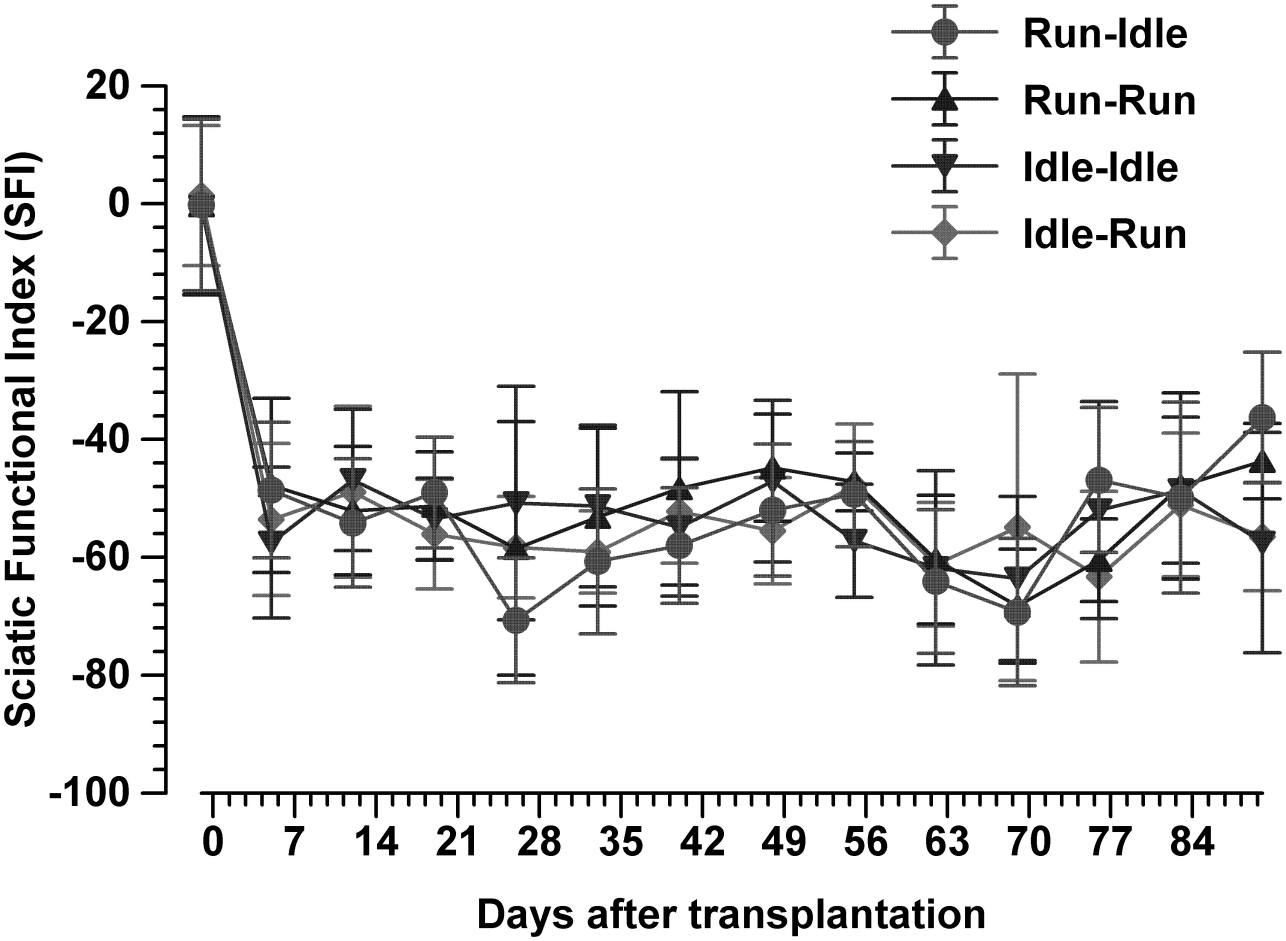
Sciatic functional index. This synopsis of the SFI of 616 walking tracks of 32 Lewis rats demonstrates no functional recovery at all. In each group, the SFI drops sharply from the normal value of 0 immediately after the sciatic nerve transplant and does not recover during 91 days of regeneration (mean ± SD, *n* = 8 rats/groups).

A total of 616 walking tracks of 32 rats were condensed into the dimensionless Sciatic Functional Index (14). In all rats, the SFI was around zero (−0.2 to 1.4) with a symmetric normal gait (Figure 2, *uppermost track*) before the lesion of the sciatic nerve. Immediately after axotomy, the total and intermediate toe spreading (ETS and EIS) on the injured side were greatly reduced in all experimental groups. The print length (EPL) was increased compared with data from the intact hind limb (NTS, NIS, and NPL). The animals severely limped with a step length from right to left (ETOF) of almost zero (Figure 2, *lower tracks*). Immediately after the graft, the SFI dropped to ~−60 (Figure 3), indicating complete paralysis of the transected nerve (14). During regeneration, the SFI should increase with the gradual restoration of muscle innervation (62); however, this did not occur as expected. The SFI remained at a low level, ranging from −35 to −69 (run–idle), −27 to −69 (run–run), −30 to −64 (idle–idle), and −16 to −63 (idle-run), as measured 13 times in a 3–91 day period after nerve reconstruction (Figure 3). There was no indication of recovery of motor function after nerve repair (Figures 2, 3). This holds true for all animals, either trained or kept idle. Repeated measure two-way ANOVA showed (1) no significant difference in SFI values during regeneration from 7 to 91 days after nerve graft and (2) no significant difference between the four training types.

### 3.4. Motor nerve conduction test measurements

The evoked compound muscle action potential on both, the right and left sides, was recorded in all experimental animals immediately before and 91 days after sciatic nerve reconstruction. In a pilot study in our laboratory, the CMAP amplitude of the soleus muscle was 16.5 ± 2.1 mV and the latency of muscle contraction was 2.1 ± 0.2 ms in six normal Lewis rats. The mean preoperative value of all 32 rats in the present study was 16.6 ± 2.5 mV amplitude and the latency was 2.3 ± 0.2 ms (average of means ± SD). Within each group, there was no apparent difference in the amplitude of CMAP and the latency of muscle contraction between the right and left sides of each animal before sciatic axotomy (Table 2).

**Table 2:** Motor nerve conduction test measurements.

	**Normal intact rats**		**91 days after nerve graft**	
**Animal groups**	**Right side**	**Left side**	**Operated side** **(right)**	**Control side** **(left)**
**Amplitude of compound muscle action potential [mV]**				
Run–idle	16.3 ± 3.6	17.2 ± 5.0	11.9 ± 4.9 (73%)	18.8 ± 7.9
Run–run	17.3 ± 4.2	17.3 ± 5.2	6.3 ± 3.4 (37%)	18.7 ± 7.8
Idle–idle	12.1 ± 3.3	13.6 ± 2.5	6.5 ± 2.2 (54%)	19.3 ± 5.6
Idle–run	18.9 ± 3.9	20.1 ± 3.9	8.1 ± 2.0 (43%)	18.0 ± 7.2
**Latency of compound muscle action potential [ms]**				
Run–idle	2.3 ± 0.7	2.1 ± 0.4	3.1 ± 0.7 (134%)	2.4 ± 0.6
Run–run	2.6 ± 0.5	2.1 ± 0.2	3.2 ± 0.6 (124%)	2.4 ± 0.7
Idle–idle	2.7 ± 0.5	2.1 ± 0.5	3.2 ± 0.6 (117%)	2.1 ± 0.5
Idle–run	2.4 ± 0.3	2.3 ± 0.5	3.1 ± 0.4 (134%)	2.0 ± 0.3

CMAP amplitude and latency of the soleus muscle were measured in the same rats immediately before the transection of the sciatic nerve and again 91 days after the nerve graft (mean ± SD, n = 8 rats/group). Data in brackets are the percent of the preoperative value.

**Figure 4:**
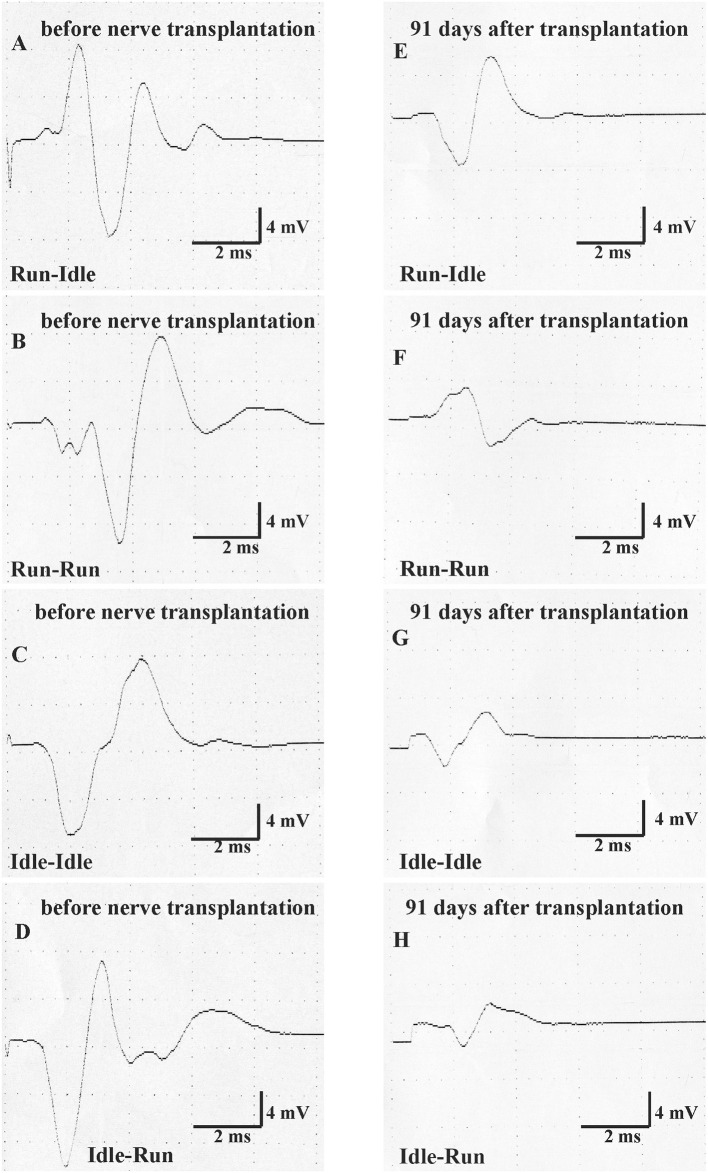
Motor nerve conduction test (MNCT). The evoked compound muscle action potential of the soleus muscle was measured first immediately before nerve transplantation in all four groups (normal CMAP, **A–D**), and second 91 days after sciatic nerve transplantation (regenerated CMAP, **E–H**).

Thirteen weeks after allograft transplantation, both CMAP amplitude and latency on the left control side were about the same as before the surgery (Table 2). On the right operated side, the amplitude in the run–idle rats reduced to 73% of the preoperative value, but this dropped to 37, 54, or 43% in the run–run, idle–idle, and idle-run, respectively (Table 2 and Figure 4). The latency of muscle contraction on the right operated side was prolonged to 117–134% of the preoperative value (Table 2).

On the left side, that is, the unoperated side repeated measure two-way ANOVA showed (1) no significant difference in CMAP amplitude and latency before and after nerve graft and (2) no significant difference between the four training types.

On the right side, that is, the side of the nerve graft the same tests showed for latency (1) a very significant difference before and after the nerve graft, but (2) no significant difference between the four training types. With regard to CMAP amplitude, the repeated measure two-way ANOVA showed (1) a very significant difference before and after the nerve graft and also (2) a significant difference between the four training types. 91 days after the nerve graft, *Post hoc* Fisher's test showed no significant differences between idle-run and the three other training groups, but rather surprisingly a significantly higher CMAP amplitude after run–idle training than after run–run (*p* = 0.012) or idle–idle (*p* = 0.011).

None of these data indicate a noticeable positive effect of treadmill training on the accuracy of muscle regeneration (Figure 1) and regain of function (Figures 2, 3)—with the exception of the increased CMAP amplitude in run–idle rats (*p* = 0.04).

## 4. Discussion

The ideal goal of any peripheral nerve reconstruction by end-to-end suture is to achieve a correct realignment of nerve fascicles and–theoretically–of the proximal and distal stumps of the same axons. While the former is very difficult (68–71), the latter appears impossible. There is no precise guide for the resprouting growth cones of ~7,100 myelinated and 51,000 non-myelinated axons comprising the regenerated sciatic nerve of an adult rat (66). Inadequate pathfinding leads to motor synkinesis, uncoordinated muscle movement, and/or sensory discrimination (36, 72). Previously, we found that the rat sciatic nerve regenerates almost as well with reconstructions delayed up to 1-year post-injury as with immediate nerve repair (5).

### 4.1. Neuronal regeneration after nerve graft

In the present study, we created a major lesion, that is, 5 mm allograft transplantation, in contrast to the crush lesion of the sciatic nerve performed in other studies (40, 43, 55). After regeneration, the number of spinal motoneurons demonstrated with retrograde labeling in the run–idle group was 94% of the normal values (549) in intact rats, 87% in each run–run and idle–idle, or 81% in idle-run. Hence, the training did not increase the number of regenerated motoneurons 13 weeks after nerve surgery—contrary to reports that are more favorable with shorter regeneration times. Molteni et al. (73) reported that after 3 days of sciatic nerve crush and regeneration, there were ~2 × as many labeled neurons in the trained mice as in the sedentary animals. English et al. (52) concluded that following transection and application of fibrin glue on the sciatic nerve, after 2 and 4 weeks of regeneration, ~120–240 labeled motoneurons were counted in treadmill-trained mice and 20–150 in untrained mice. Overall, these data sets, in combination with ours, suggest that training “enhances” or “accelerates” axon regeneration in the early phase of regeneration but does not improve the final functional outcome.

### 4.2. Accuracy of muscle reinnervation and assessment of functional outcome

In our study, the quantitative regrowth of motoneuron axons distal to the sciatic nerve graft through the soleus and common fibular nerves was distinctly successful in all rats after nerve repair. However, the qualitative accuracy of reinnervation (Figure 1) and the recovery of motor function (Figures 2, 3) proved unsuccessful. The functional outcome, judged by SFI, was equally poor in all rats, run–idle, idle-run, run–run, or idle–idle. Neither allograft nerve graft (present study), end-to-end nerve suture, or artificial nerve conduit have achieved correct myotopic reinnervation (2, 3, 7, 31–34). Even cut and immediate suture of the thin (< 0.2 mm) buccal branch of the facial nerve in rats in close proximity to its target muscle causes severe misdirection of reinnervation (34). The greatest benefit of motor reinnervation is the restoration of muscle tonus and the reduction of muscle atrophy.

In contrast to our present experiment, a crush lesion of the nerve is a relatively minor injury that does not change the myotopic organization of motoneurons in rats (3) and allows full recovery of the SFI (74). After crush axons presumably resprout rather correctly along with the bands of Büngner (39) back to their original targets due to the guidance of basal lamina tubes (68). Nerve transection, however, interrupts all basal lamina tubes, leads to the mis-sprouting of axonal branches, and destroys myotopic reinnervation (Figure 1) that cannot be compensated by central plasticity (75). In particular, double-labeled neurons, that were never observed in intact animals, simultaneously innervate fibers of the flexor and extensor muscles, and thus cause an antagonistic inhibition, also referred to as auto- or post-paralytic syndrome (3–6, 24, 27, 28, 76).

In several regeneration studies after sciatic nerve crush, forced or voluntary exercise has been reported to improve functional recovery, enhance the return of a sensorimotor function (40), increase sensory axon regeneration (73), promote remyelination of the injured nerve (77), and enlarge myofibril cross-sectional areas and diminish collagen around muscle fibers (46). Staircase training has improved task-specific performance as well as nonspecific motor and sensorimotor activities following the contusion of the rat spinal cord (78). Our long-term data after nerve graft that entail complete transection of all nerve fibers greatly differ from these more favorable results after sciatic nerve crush (40, 46, 73, 77). But why? If our training regime had negatively influenced regeneration, the animals in the idle–idle and the run–idle groups should have yielded better outcomes than run–run and idle-run rats. This was definitively not the case (Figure 3 and Table 2). Our neuronal labeling data prove that neural regeneration was complete and poor functional recovery due to neither lack of repair [compare also (79)] nor overt errors during the procedure. Hence, treadmill training, apparently independent of stress, does not alter the recovery of function after complete transection and regeneration of the sciatic nerve. The only favorable new data of the present study is the beneficial effect of preoperative training on the CMAP of the reinnervated muscle. The amplitude of CMAP was about 16.4 mV in healthy intact rats. This value was dramatically decreased and the difference was statistically significant after 91 days of regeneration in all experimental groups with the exception of the run–idle group. The run–idle group exhibited better contractility and strength of the soleus muscle compared with other groups. Our data prove that physical fitness due to exercise before nerve injury has a beneficial effect on healing—indeed a better effect than physical exercise during regeneration.

## 5. Conclusion

Our data confirm with long observation times, ensuring complete regeneration, that treadmill training after sciatic nerve injury has neither beneficial nor harmful effects on muscle reinnervation and recovery of motor function. We obtained no evidence that treadmill training improves the accuracy of reinnervation.

## Data availability statement

The raw data supporting the conclusions of this article will be available by the corresponding author, without undue reservation.

## Ethics statement

The experimental animal study was reviewed and approved by the Bezirksregierung Köln (Az. 50.203.2-K35, 34/2001) based on the guidelines of the European Union Council (86/609/EU) and according to § 8 Tierschutzgesetz (German Federal Law for the Protection of Animals). Written informed consent was obtained from the owners for the participation of their animals in this study.

## Author contributions

MB, JA, and WN: study concept and design. MB, JA, and HM: data acquisition. MB, HM, US, JV, and WN: analysis and interpretation of data. MB and JV: drafting of the manuscript. JV, US, and WN: critical revision of the manuscript for important intellectual content. US and WN: obtained funding. WN: study supervision. All authors had full access to all the data in the study and take responsibility for the integrity of the data and the accuracy of the data analysis. All authors contributed to the article and approved the submitted version.

## Supplementary material

Supplementary Figure 1 Footprints. The left side **(A)** shows the footprints of a normal, intact rat 1 day before sciatic nerve graft; the right side **(B)** shows the footprints of a rat 13 weeks after 5-mm sciatic nerve graft on the right experimental side. EIS, Experimental Intermediary Toe-Spreading; EPL, Experimental Print Length; ETOF, Experimental To Opposite Foot; ETS, Experimental Total Toe-Spreading; NIS, Normal Intermediary Toe-Spreading; NPL, Normal Print Length; NTOF, Normal To Opposite Foot; NTS, Normal Total Toe-Spreading.

## References

[1] Meek MF Den Dunnen WF Schakenraad JM Robinson Robinson PH: Long-term evaluation of functional nerve recovery after reconstruction with a thin-walled biodegradable poly (DL-lactide-epsilon-caprolactone) nerve guide using walking track analysis and electrostimulation tests. Microsurgery. (1999) 19:247–253. 10.1002/(SICI)1098-2752(1999)19:5<247::AID-MICR7>3.0.CO;2-E 10413791

[2] Valero-Cabré A Navarro X. Functional impact of axonal misdirection after peripheral nerve injuries followed by graft or tube repair. J Neurotrauma. (2002) 19:1475–85. 10.1089/089771502320914705 12490012

[3] Valero-Cabré A Tsironis K Skouras E Navarro X Neiss WF. Peripheral and spinal motor reorganization after nerve injury and repair. J Neurotrauma. (2004) 21:95–108. 10.1089/089771504772695986 14987469

[4] Valero-Cabré A Tsironis K Skouras E Perego G Navarro X Neiss WF: Superior muscle reinnervation after autologous nerve graft or poly-L-lactide-epsilon-caprolactone (PLC) tube implantation in comparison to silicone tube repair. J Neurosci Res. (2001) 63:214–223. 10.1002/1097-4547(20010115)63:2<214::AID-JNR1014>3.0.CO;2-D 11169632

[5] Barham M Andermahr J Lee J-I Neiss WF. Successful reinnervation but poor recovery of motor function following sciatic nerve transplant delayed up to one year after axotomy in rats. Int J Neuroprotect Neuroregener. (2007) 3:225–38.

[6] Barham M Streppel M Guntinas-Lichius O Fulgham-Scott N Vogt J Neiss WF. Treatment with nimodipine or FK506 after facial nerve repair neither improves accuracy of reinnervation nor recovery of mimetic function in rats. Front Neurosci. (2022) 16:895076. 10.3389/fnins.2022.895076 35645727 PMC9136327

[7] Hamilton SK Hinkle ML Nicolini J Rambo LN Rexwinkle AM Rose SJ. Misdirection of regenerating axons and functional recovery following sciatic nerve injury in rats. J Comp Neurol. (2011) 519:21–33. 10.1002/cne.22446 21120925 PMC3703664

[8] Udina E Cobianchi S Allodi I Navarro X. Effects of activity-dependent strategies on regeneration and plasticity after peripheral nerve injuries. Ann Anat. (2011) 193:347–53. 10.1016/j.aanat.2011.02.012 21514121

[9] Udina E Puigdemasa A Navarro X. Passive and active exercise improve regeneration and muscle reinnervation after peripheral nerve injury in the rat. Muscle Nerve. (2011) 43:500–9. 10.1002/mus.21912 21305568

[10] Jang SH Lee JH. Effects of physical exercise on the functional recovery of rat hindlimbs with impairments of the sciatic nerve as assessed by 2D video analysis. J Phys Ther Sci. (2015) 27:935–8. 10.1589/jpts.27.935 25931763 PMC4395747

[11] Catapano J Willand MP Zhang JJ Scholl D Gordon T Borschel GH. Retrograde labeling of regenerating motor and sensory neurons using silicone caps. J Neurosci Methods. (2016) 259:122–8. 10.1016/j.jneumeth.2015.11.020 26658222

[12] Mackinnon SE Hudson AR Hunter DA. Histologic assessment of nerve regeneration in the rat. Plast Reconstr Surg. (1985) 75:384–8. 10.1097/00006534-198503000-00014 2579408

[13] Bain JR Mackinnon SE Hunter DA. Functional evaluation of complete sciatic, peroneal, and posterior tibial nerve lesions in the rat. Plast Reconstr Surg. (1989) 83:129–38. 10.1097/00006534-198901000-00025 2909054

[14] de Medinaceli L Freed WJ Wyatt RJ. An index of the functional condition of rat sciatic nerve based on measurements made from walking tracks. Exp Neurol. (1982) 77:634–43. 10.1016/0014-4886(82)90234-5 7117467

[15] de Medinaceli L Leblanc AL Merle M. Functional consequences of isolated nerve stretch: experimental long-term static loading. J Reconstr Microsurg. (1997) 13:185–92. 10.1055/s-2007-1006403 9101448

[16] Hare GM Evans PJ Mackinnon SE Best TJ Bain JR Szalai JP. Walking track analysis: a long-term assessment of peripheral nerve recovery. Plast Reconstr Surg. (1992) 89:251–8. 10.1097/00006534-199202000-00009 1732892

[17] Ilha J Araujo RT Malysz T Hermel EE Rigon P Xavier LL. Endurance and resistance exercise training programs elicit specific effects on sciatic nerve regeneration after experimental traumatic lesion in rats. Neurorehabil Neural Repair. (2008) 22:355–66. 10.1177/1545968307313502 18326889

[18] Casal D Mota-Silva E Iria I Pais D Farinho A Alves S. Functional and physiological methods of evaluating median nerve regeneration in the rat. J Vis Exp. (2020) 158:e59767. 10.3791/59767 32364547

[19] Hruska RE Kennedy S Silbergeld EK. Quantitative aspects of normal locomotion in rats. Life Sci. (1979) 25:171–9. 10.1016/0024-3205(79)90389-8 491843

[20] Westerga J Gramsbergen A. The development of locomotion in the rat. Brain Res Dev Brain Res. (1990) 57:163–74. 10.1016/0165-3806(90)90042-W 2073717

[21] Dijkstra JR Meek MF Robinson PH Gramsbergen A. Methods to evaluate functional nerve recovery in adult rats: walking track analysis, video analysis and the withdrawal reflex. J Neurosci Methods. (2000) 96:89–96. 10.1016/S0165-0270(99)00174-0 10720672

[22] Rui J Runge MB Spinner RJ Yaszemski MJ Windebank AJ Wang H. Gait cycle analysis: parameters sensitive for functional evaluation of peripheral nerve recovery in rat hind limbs. Ann Plast Surg. (2014) 73:405–11. 10.1097/SAP.0000000000000008 24317246

[23] Langley JN Hashimoto M. On the suture of separate nerve bundles in a nerve trunk and on internal nerve plexuses. J Physiol. (1917) 51:318–46. 10.1113/jphysiol.1917.sp001805 16993388 PMC1402701

[24] Esslen E. Electromyographic findings on two types of misdirection of regenerating axons. Electroencephalogr Clin Neurophysiol. (1960) 12:738–41. 10.1016/0013-4694(60)90120-6 13820840

[25] Brushart TM. Preferential reinnervation of motor nerves by regenerating motor axons. J Neurosci. (1988) 8:1026–31. 10.1523/JNEUROSCI.08-03-01026.1988 3346713 PMC6569224

[26] Brushart TM Seiler WA. Selective reinnervation of distal motor stumps by peripheral motor axons. Exp Neurol. (1987) 97:289–300. 10.1016/0014-4886(87)90090-2 3609213

[27] Angelov DN Skouras E Guntinas-Lichius O Streppel M Popratiloff A Walther M. Contralateral trigeminal nerve lesion reduces polyneuronal muscle innervation after facial nerve repair in rats. Eur J Neurosci. (1999) 11:1369–78. 10.1046/j.1460-9568.1999.00545.x 10103132

[28] Dohm S Streppel M Guntinas-Lichius O Pesheva P Probstmeier R Walther M. Local application of extracellular matrix proteins fails to reduce the number of axonal branches after varying reconstructive surgery on rat facial nerve. Restor Neurol Neurosci. (2000) 16:117–26. 12671214

[29] Udina E Rodriguez FJ Verdu E Espejo M Gold BG Navarro X. FK506 enhances regeneration of axons across long peripheral nerve gaps rapaired with collagen guides seeded with allogeneic Schwann cells. Glia. (2004) 472:120–9. 10.1002/glia.20025 15185391

[30] de Ruiter GC Malessy MJ Alaid AO Spinner RJ Engelstad JK Sorenson EJ. Misdirection of regenerating motor axons after nerve injury and repair in the rat sciatic nerve model. Exp Neurol. (2008) 211:339–50. 10.1016/j.expneurol.2007.12.023 18448099 PMC2967197

[31] Sabatier MJ To BN Nicolini J English AW. Effect of axon misdirection on recovery of electromyographic activity and kinematics after peripheral nerve injury. Cells Tissues Organs. (2011) 193:298–309. 10.1159/000323677 21411964 PMC3128140

[32] de Ruiter GC Spinner RJ Verhaagen J Malessy MJ. Misdirection and guidance of regenerating axons after experimental nerve injury and repair. J Neurosurg. (2014) 120:493–501. 10.3171/2013.8.JNS122300 24116727

[33] Ozsoy U Demirel BM Hizay A Ozsoy O Ankerne J Angelova S. Manual stimulation of the whisker pad after hypoglossal-facial anastomosis (HFA) using a Y-tube conduit does not improve recovery of whisking function. Exp Brain Res. (2014) 232:2021–33. 10.1007/s00221-014-3892-2 24623354

[34] Harris GR Breazzano MP Shyu I Donahue SP Lavin PJM. Oculomotor Synkinesis (Aberrant Reinnervation of the Third Cranial Nerve) Associated with Atypical Tolosa-Hunt Syndrome. Neuroophthalmology. (2019) 44:262–6. 10.1080/01658107.2019.1576738 33012913 PMC7518316

[35] Montserrat L Benito M. Facial synkinesis and aberrant regeneration of facial nerve. Adv Neurol. (1988) 49:211–24. 3278542

[36] Sumner AJ. Aberrant reinnervation. Muscle Nerve. (1990) 13:801–3. 10.1002/mus.880130905 2233866

[37] Guntinas-Lichius O Glowka TR Angelov DN Irintchev A Neiss WF. Improved functional recovery after facial nerve reconstruction by temporary denervation of the contralateral mimic musculature with botulinum toxin in rats. Neurorehabil Neural Repair. (2011) 25:15–23. 10.1177/1545968310376058 20930211

[38] Seitz M Grosheva M Skouras E Angelova SK Ankerne J Jungnickel J. Poor functional recovery and muscle polyinnervation after facial nerve injury in fibroblast growth factor-2-/- mice can be improved by manual stimulation of denervated vibrissal muscles. Neuroscience. (2011) 182:241–7. 10.1016/j.neuroscience.2011.03.032 21440044

[39] Büngner Ov: Über die Degenerations- und Regenerationsvorgänge am Nerven nach Verletzungen. Beiträge zur Pathologischen Anatomie und zur Pathologie. (1891) 10:321–93

[40] van Meeteren NL Brakkee JH Hamers FP Helders PJ Gispen WH. Exercise training improves functional recovery and motor nerve conduction velocity after sciatic nerve crush lesion in the rat. Arch Phys Med Rehabil. (1997) 78:70–7. 10.1016/S0003-9993(97)90013-7 9014961

[41] Milicin C Sîrbu E. A comparative study of rehabilitation therapy in traumatic upper limb peripheral nerve injuries. Neuro Rehabil. (2018) 42:113–9. 10.3233/NRE-172220 29400678

[42] Seo TB Han IS Yoon JH Hong KE Yoon SJ Namgung U. Involvement of Cdc2 in axonal regeneration enhanced by exercise training in rats. Med Sci Sports Exerc. (2006) 38:1267–76. 10.1249/01.mss.0000227311.00976.68 16826023

[43] Bonetti LV Korb A Da Silva SA Ilha J Marcuzzo S Achaval M. Balance and coordination training after sciatic nerve injury. Muscle Nerve. (2011) 44:55–62. 10.1002/mus.21996 21488054

[44] Bobinski F Martins DF Bratti T Mazzardo-Martins L Winkelmann-Duarte EC Guglielmo LG. Neuroprotective and neuroregenerative effects of low-intensity aerobic exercise on sciatic nerve crush injury in mice. Neuroscience. (2011) 194:337–48. 10.1016/j.neuroscience.2011.07.075 21864654

[45] Boeltz T Ireland M Mathis K Nicolini J Poplavski K Rose SJ. Effects of treadmill training on functional recovery following peripheral nerve injury in rats. J Neurophysiol. (2013) 109:2645–57. 10.1152/jn.00946.2012 23468390 PMC3680800

[46] Bonetti LV Malysz T Ilha J Barbosa S Achaval M Faccioni-Heuser MC. The effects of two different exercise programs on the ultrastructural features of the sciatic nerve and soleus muscle after sciatic crush. Anat Rec (Hoboken). (2017) 300:1654–61. 10.1002/ar.23611 28463452

[47] Bonetti LV Schneider AP Barbosa S Ilha J Faccioni-Heuser MC. Balance and coordination training and endurance training after nerve injury. Muscle Nerve. (2015) 51:83–91. 10.1002/mus.24268 24752648

[48] van Meeteren NL Brakkee JH Helders PJ Gispen WH. The effect of exercise training on functional recovery after sciatic nerve crush in the rat. J Peripher Nerv Syst. (1998) 3:277–82. 10970128

[49] Marqueste T Alliez JR Alluin O Jammes Y Decherchi P. Neuromuscular rehabilitation by treadmill running or electrical stimulation after peripheral nerve injury and repair. J Appl Physiol. (2004) 96:1988–95. 10.1152/japplphysiol.00775.2003 14634028

[50] Sabatier MJ Redmon N Schwartz G English AW. Treadmill training promotes axon regeneration in injured peripheral nerves. Exp Neurol. (2008) 211:489–93. 10.1016/j.expneurol.2008.02.013 18420199 PMC2584779

[51] Asensio-Pinilla E Udina E Jaramillo J Navarro X. Electrical stimulation combined with exercise increase axonal regeneration after peripheral nerve injury. Exp Neurol. (2009) 219:258–65. 10.1016/j.expneurol.2009.05.034 19500575

[52] English AW Cucoranu D Mulligan A Sabatier M. Treadmill training enhances axon regeneration in injured mouse peripheral nerves without increased loss of topographic specificity. J Comp Neurol. (2009) 517:245–55. 10.1002/cne.22149 19731339 PMC2804895

[53] English AW Wilhelm JC Sabatier MJ. Enhancing recovery from peripheral nerve injury using treadmill training. Ann Anat. (2011) 193:354–61. 10.1016/j.aanat.2011.02.013 21498059 PMC3137663

[54] Sinis N Guntinas-Lichius O Irintchev A Skouras E Kuerten S Pavlov SP. Manual stimulation of forearm muscles does not improve recovery of motor function after injury to a mixed peripheral nerve. Exp Brain Res. (2008) 185:469–83. 10.1007/s00221-007-1174-y 17955222

[55] de Oliveira Marques C Amaro Espindula I Kwame Karikari Darko E Viçosa Bonetti L Sonza A Aparecida Partata W. Whole-body vibration therapy does not improve the peripheral nerve regeneration in experimental model. J Musculoskelet Neuronal Interact. (2021) 21:68–78. 33657756 PMC8020024

[56] Rustemeyer J Krajacic A Dicke U. Histomorphological and functional impacts of postoperative motor training in rats after allograft sciatic nerve transplantation under low-dose FK 506. Muscle Nerve. (2009) 39:480–8. 10.1002/mus.21251 19260056

[57] Carr MM Best TJ Mackinnon SE Evans PJ. Strain differences in autotomy in rats undergoing sciatic nerve transection or repair. Ann Plast Surg. (1992) 28:538–44. 10.1097/00000637-199228060-00008 1622035

[58] Chamberlain LJ Yannas IV Hsu HP Strichartz GR Spector M. Near-terminus axonal structure and function following rat sciatic nerve regeneration through a collagen-GAG matrix in a ten-millimeter gap. J Neurosci Res. (2000) 60:666–77. 10.1002/(SICI)1097-4547(20000601)60:5&lt 10820438

[59] Klapdor K Dulfer BG Hammann A Van der Staay FJ. A low-cost method to analyse footprint patterns. J Neurosci Methods. (1997) 75:49–54. 10.1016/S0165-0270(97)00042-3 9262143

[60] Cação-Benedini LO Ribeiro PG Gomes AR Ywazaki JL Monte-Raso VV Prado CM. Remobilization through stretching improves gait recovery in the rat. Acta Histochem. (2013) 115:460–9. 10.1016/j.acthis.2012.11.001 23265777

[61] Johnston RB Zachary L Dellon AL. Seiler WAt, Teplica DM: Improved imaging of rat hindfoot prints for walking track analysis. J Neurosci Methods. (1991) 38:111–4. 10.1016/0165-0270(91)90161-R 1784117

[62] de Medinaceli L Seaber AV. Experimental nerve reconnection: importance of initial repair. Microsurgery. (1989) 10:56–70. 10.1002/micr.1920100111 2725257

[63] Greene EC. Anatomy of the rat. Transac Am Philos Soc New Ser. (1935) 27:1–370. 10.2307/1005513

[64] Schmalbruch H: Fiber composition of the rat sciatic nerve. Anat Rec. (1986) 215:71–81. 10.1002/ar.1092150111 3706794

[65] Gundersen HJ: Stereology of arbitrary particles. A review of unbiased number and size estimators and the presentation of some new ones, in memory of William R. Thompson. J Microsc. (1986) 143:3–45 10.1111/j.1365-2818.1986.tb02764.x 3761363

[66] Auer M Allodi I Barham M Udina E Neiss WF Navarro X. C3 exoenzyme lacks effects on peripheral axon regeneration in vivo. J Peripher Nerv Syst. (2013) 18:30–6. 10.1111/jns5.12004 23521641

[67] Neiss WF Guntinas Lichius O Angelov DN Gunkel A Stennert E. The hypoglossal-facial anastomosis as model of neuronal plasticity in the rat. Ann Anat. (1992) 174:419–33. 10.1016/S0940-9602(11)80266-9 1449219

[68] Lundborg G. Nerve Injury and Repair: Regeneration, Reconstruction, and Cortical Remodeling 2nd edn. Philadelphia: Elsevier/Churchill Livingstone (2004). p. 248.

[69] Evans PJ Bain JR Mackinnon SE Makino AP Hunter DA. Selective reinnervation: a comparison of recovery following microsuture and conduit nerve repair. Brain Res. (1991) 559:315–21. 10.1016/0006-8993(91)90018-Q 1794104

[70] Millesi H. Chirurgie der peripheren Nerven. München, Wien, Baltimore: Urban & Schwarzenberg (1992). p. 232.

[71] Amara B de Medinaceli L Lane GB Merle M. Functional assessment of misdirected axon growth after nerve repair in the rat. J Reconstr Microsurg. (2000) 16:563–7. 10.1055/s-2000-8396 11083397

[72] Aldskogius H Molander C. Specificity in regenerative outgrowth and target reinnervation by mammalian peripheral axons. Restor Neurol Neurosci. (1990) 1:275–80. 10.3233/RNN-1990-13415 21551567

[73] Molteni R Zheng JQ Ying Z Gómez-Pinilla F Twiss JL. Voluntary exercise increases axonal regeneration from sensory neurons. Proc Natl Acad Sci U S A. (2004) 101:8473–8. 10.1073/pnas.0401443101 15159540 PMC420418

[74] Malysz T Ilha J Nascimento PS De Angelis K Schaan BD Achaval M. Beneficial effects of treadmill training in experimental diabetic nerve regeneration. Clinics (São Paulo). (2010) 65:1329–37. 10.1590/S1807-59322010001200017 21340223 PMC3020345

[75] Gruart A Streppel M Guntinas-Lichius O Angelov DN Neiss WF Delgado-García JM. Motoneuron adaptability to new motor tasks following two types of facial-facial anastomosis in cats. Brain. (2003) 126:115–133. 10.1093/brain/awg008 12477700

[76] Akulov MA Orlova OR Tabashnikova TV Karnaukhov VV Orlova AS. Facial nerve injury in neurosurgery: a rehabilitation potential of botulinum therapy. Zh Vopr Neirokhir Im N N Burdenko. (2018) 82:111–8. 10.17116/neiro2018821111-118 29543223

[77] Kim J-O Seo T Yoon J-H. Effects of treadmill training on axonal regeneration, spinal cord motor neuron, GAP-43 & GLUT-4 protein expression after sciatic nerve injury in the streptozotocin-induced diabetic rats. Int J Applied Sports Sci. (2007) 19:117–33.

[78] Singh A Murray M Houle JD. A training paradigm to enhance motor recovery in contused rats: effects of staircase training. Neurorehabil Neural Repair. (2011) 25:24–34. 10.1177/1545968310378510 20858910

[79] Streppel M Angelov DN Guntinas-Lichius O Hilgers RD Rosenblatt JD Stennert E. Slow axonal regrowth but extreme hyperinnervation of target muscle after suture of the facial nerve in aged rats. Neurobiol Aging. (1998) 19:83–8. 10.1016/S0197-4580(97)00163-2 9562508

